# Federated Abnormal Heart Sound Detection with Weak to No Labels

**DOI:** 10.34133/cbsystems.0152

**Published:** 2024-09-10

**Authors:** Wanyong Qiu, Chen Quan, Yongzi Yu, Eda Kara, Kun Qian, Bin Hu, Björn W. Schuller, Yoshiharu Yamamoto

**Affiliations:** ^1^ Key Laboratory of Brain Health Intelligent Evaluation and Intervention, Ministry of Education (Beijing Institute of Technology), Beijing 100081, China.; ^2^ School of Medical Technology and School of Computer Science, Beijing Institute of Technology, Beijing 100081, China.; ^3^ CHI—Chair of Health Informatics, MRI, Technical University of Munich, 80290 Munich, Germany.; ^4^ GLAM—Group on Language, Audio, & Music, Imperial College London, London SW7 2AZ, UK.; ^5^ Edicational Physiology Laboratory, Graduate School of Education, The University of Tokyo, Tokyo 113-0033, Japan.

## Abstract

Cardiovascular diseases are a prominent cause of mortality, emphasizing the need for early prevention and diagnosis. Utilizing artificial intelligence (AI) models, heart sound analysis emerges as a noninvasive and universally applicable approach for assessing cardiovascular health conditions. However, real-world medical data are dispersed across medical institutions, forming “data islands” due to data sharing limitations for security reasons. To this end, federated learning (FL) has been extensively employed in the medical field, which can effectively model across multiple institutions. Additionally, conventional supervised classification methods require fully labeled data classes, e.g., binary classification requires labeling of positive and negative samples. Nevertheless, the process of labeling healthcare data is time-consuming and labor-intensive, leading to the possibility of mislabeling negative samples. In this study, we validate an FL framework with a naive positive-unlabeled (*PU*) learning strategy. Semisupervised FL model can directly learn from a limited set of positive samples and an extensive pool of unlabeled samples. Our emphasis is on vertical-FL to enhance collaboration across institutions with different medical record feature spaces. Additionally, our contribution extends to feature importance analysis, where we explore 6 methods and provide practical recommendations for detecting abnormal heart sounds. The study demonstrated an impressive accuracy of 84%, comparable to outcomes in supervised learning, thereby advancing the application of FL in abnormal heart sound detection.

## Introduction

Cardiovascular diseases (CVDs) are the leading cause of death worldwide, surpassing other causes in annual fatalities [1,2]. The importance of early diagnosis and preventive measures in cardiovascular healthcare cannot be overstressed. Due to its universal and noninvasive nature, heart sound analysis offers a promising avenue in medical care for assessing an individual’s cardiovascular status. Leveraging machine learning models for abnormal heart sound detection in digital healthcare provides a practical approach for early diagnosis and effective prevention of CVDs [3–6].

However, the issues of privacy protection and data silos seriously impede the exploration of medical data and the application of medical artificial intelligence (AI) models [7]. First, variations exist among medical institutions. Some institutions have limited resources and records that hinder effective medical machine learning modeling. Second, pertinent laws and regulations, including the Health Insurance Portability and Accountability Act (HIPAA) [8], restrict data exchange between medical institutions for security and privacy protection. Consequently, healthcare data become fragmented and scattered across medical institutions, causing the phenomenon of “data islands.”

Federated learning (FL) is a distributed machine learning paradigm that enables collaborative modeling among participants without sharing their private data [9–11]. It serves as a viable method to address the “data island” issue in the medical field through collaborative modeling across multiple centers. Consequently, it provides a certain degree of protection for data security and patient privacy. Our studies are based on *SecureBoost* [12], a federated ensemble learning framework embedded in FATE. [FATE (Federated AI Technology Enabler [13]) supports the FL architecture, as well as the secure computation and development of various machine learning algorithms; https://github.com/FederatedAI/FATE.] In this study, we practically applied the vertical-*SecureBoost* (Vertically Federated XGBoost) model on a multi-institutional heart sound database. [XGBoost (eXtreme Gradient Boosting [14]) provides an optimized distributed gradient boosting tree-based ensemble model designed to be highly efficient, flexible, and portable; https://xgboost.readthedocs.io.] We propose corresponding federated optimization strategies for the requirements of real-world healthcare scenarios with label scarcity.

In real-life medical scenarios, we consider 3 key issues: (a) Accurately labeling all heart sound records is resource-intensive, leading to only a fraction of the dataset being labeled [15–17]. Semisupervised FL is considered suitable, involving a few “positive” labeled samples and a large volume of “unlabeled” samples, which may contain both positive and negative samples. (b) The widely studied horizontal-FL, also known as sample-partitioned FL [18], requires data from institutions to have the same feature space and different sample spaces. Horizontal-FL is devised to facilitate collaboration among medical institutions with varied patient populations, given the inability to share data across institutions. Therefore, horizontal-FL data partitioning is recommended when developing models with limited sample size variability of FL participants. However, in real medical scenarios, the same patient may receive treatment at different hospitals, allowing for the use of records from multiple sources in diagnosis. Consequently, multiple healthcare institutions may serve the same patient population. Vertical-FL, akin to feature-partitioned FL [19], has recently garnered attention from researchers in cases where medical institutions participating in FL share the same user community but have different medical record feature spaces. This study centers on vertical-FL, aiming to model collaboration across multiple institutions with distinct medical record spaces to provide comprehensive insights into the same patient population. (c) Leveraging the high-dimensional features extracted from heart sound records, it is necessary to select an effective feature importance analysis scheme to retain the most influential feature set [20]. This enhances the efficiency of FL modeling and is anticipated to sustain comparable performance while achieving a reduction in feature dimensionality. Therefore, the contributions of our work can be summarized as follows:

• Our study uniquely shifts from traditional data-centric centralized learning to embrace the FL paradigm in the analysis of the PhysioNet/CinC heart sound database. (Classification of Normal/Abnormal Heart Sound Recordings [21,22]: the PhysioNet/Computing in Cardiology Challenge; https://physionet.org/content/challenge-2016/1.0.0.) We adopt a vertical data partitioning approach and leverage the vertical-*SecureBoost* FL framework for multi-medical center collaboration modeling to address data islands and privacy concerns in healthcare.

• To meet the demands of real medical scenarios, we promote an FL framework with a naive positive-unlabeled (*PU*) semisupervised learning strategy. In specific medical contexts, semisupervised FL emphasizes the integration of positive and unlabeled training strategies. The approach achieves a remarkable 84% accuracy, comparable to the outcomes of supervised learning, representing an important exploration of FL in the realm of abnormal heart sound detection.

• In our study practice, we explore 6 distinct methods for feature importance analysis. Utilizing the ensemble learning paradigm based on XGBoost, we compare 5 methods, namely, “*gain*, *total_gain*, *cover*, *total_cover*, *weight*,” with the *SHAP* method. [SHAP (SHapley Additive exPlanations [23]) is a game-theoretic method to explain the output of machine learning models. The method is used to determine the importance of an individual by calculating the contribution of that individual in the cooperation; https://shap.readthedocs.io.] Based on comparative experiments, we provide practical recommendations for feature selection in the context of abnormal heart sound detection.

The rest of the paper is organized as follows: The “Related Works” section introduces the related work. The “Materials and Methods” section describes data preprocessing methods, experimental design, and evaluation metrics. The “Experiment and Results” section presents our comparative experiments and results. The “Discussion” section provides a detailed discussion. Finally, we conclude the paper in the “Conclusion” section. 

### Related works

In the realm of healthcare, FL has emerged as a pivotal research area, addressing the challenges associated with collaborative modeling across diverse medical institutions. Recent studies emphasize its application in multicenter settings, enabling model training without raw data exchange, thus preserving privacy and adhering to data security regulations. Researchers have investigated federated approaches for tasks such as predictive modeling, disease diagnosis, and personalized treatment recommendations. Examples of noteworthy work include the following:

• Privacy-preserving patient data sharing: Pioneering studies have focused on preserving patient privacy while enabling collaborative model training [24,25]. Techniques such as federated averaging and secure aggregation have been employed to facilitate model updates without raw data sharing. This ensures that FL complies with data protection regulations such as HIPAA.

• Decentralized disease prediction models: Some researchers have applied FL to construct disease prediction models using data across multiple healthcare institutions [26–28]. This approach allows each institution to contribute to the model without sharing patient-specific information, enabling the development of robust and generalizable models.

• Real-world federated systems: Emerging research involves the implementation of FL systems in real-world healthcare settings [29–31]. These systems consider challenges like data heterogeneity, communication efficiency, and model convergence across multiple institutions.

A practical concern often overlooked in healthcare is the limited availability of labeled data. We study the real-world setting of FL medical applications, where assuming fully labeled data in each FL client is less practical. Two related areas include federated unsupervised representation learning and federated semisupervised learning. In scenarios with limited labeled data, semisupervised FL becomes crucial [15–17]. This paradigm involves training models using a combination of labeled and unlabeled data, making it particularly relevant for medical applications with limited annotated datasets. In terms of semisupervised FL, some studies explore cross-institutional transfer learning strategies to transfer knowledge between institutions with varying degrees of labeled data [32]. Therefore, models can leverage labeled data from one institution to enhance the performance on different institution datasets, contributing to better generalization. Additionally, some studies incorporate active learning techniques within FL frameworks to intelligently select and query instances for annotation [33]. This ensures efficient utilization of labeling resources and enhances model performance in scenarios with limited labeled samples. There are few FL studies directly addressing federated *PU* learning. Study [34] proposes a novel framework called Federated Learning with Positive and Unlabeled Data (FedPU). FedPU considers that each client can label only a limited amount of data for some classes. The work [35] introduces the FedMatch algorithm, a state-of-the-art federated semisupervised model based on consistency regularization training. FedMatch addresses scenarios where clients have both labeled and unlabeled data. We study the problem of learning from positive and unlabeled (*PU*) data in the federated setting. In contrast to the previous scenario, we focus on situations where some clients exclusively have positive and unlabeled samples, while others have only unlabeled samples.

To sum up, FL in healthcare is developing rapidly, with a focus on preserving privacy and addressing data distribution challenges. The incorporation of semisupervised learning techniques further extends the applicability of federated approaches, especially in scenarios with imbalanced or limited labeled data. These developments set the stage for tackling complex tasks like abnormal heart sound detection across multiple federated care institutions.

## Materials and Methods

### Dataset description and preprocessing

**Fig. 1.**
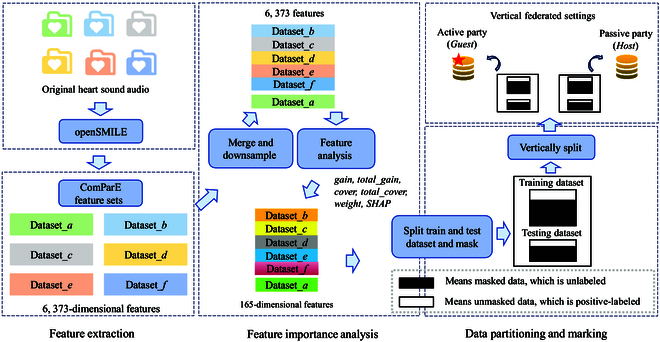
Illustration of the data preprocessing process.

In this work, heart sound data are obtained from the PhysioNet/CinC [21,22] challenge, a high-quality, authentic public database. As shown in Table 1, it comprises 6 sub-databases, each independently gathered by diverse institutions in clinical and nonclinical environments. Samples labeled as “normal” originate from healthy subjects, whereas “abnormal” samples are derived from patients with various conditions like heart valve disease and coronary artery disease. We use openSMILE [36,37], a widely used open-source toolkit for audio-signal processing, to extract features. openSMILE provides features commonly used in traditional acoustic signal processing methods, including mel frequency cepstrum coefficients (MFCCs), physiological acoustic features, and energy spectrum features. Initially, it extracts low-level descriptor (LLD) features from the audio signal and then re-extracts statistical features from these frame-based LLD features. We use the ComParE [38] feature set in openSMILE, extracting a total of 6,373 dimensional features, which include 65 acoustic LLD features and their associated statistical features. The data preprocessing procedure is summarized in Fig. 1, and the specific steps are outlined below.

**Table 1.** Summary of the sub-databases used in the PhysioNet/CinC Challenge

Sub-set/source	Recordings	# Raw recordings				# Recording length (s)		
		Abnormal/proportion (%)		Normal/proportion (%)		Min	Median	Max
Dataset_a_ (MIT)	409	292	67.5	117	28.4	9.3	35.6	36.5
Dataset_b_ (AAD)	490	104	14.9	386	60.2	5.3	8	8
Dataset_c_ (AUTH)	31	24	64.5	7	22.6	9.6	44.4	122.0
Dataset_d_ (UHA)	55	28	47.3	27	47.3	6.6	12.3	48.5
Dataset_e_ (DLUT)	2,141	183	7.1	1,958	86.7	8.1	21.1	101.7
Dataset_f_ (SUA)	114	34	27.2	80	68.4	29.4	31.7	59.6
Validation	300	150	50.0	150	50.0	5.3	21.1	122.0
Total/average	3,240	665	18.1	2,575	73.0	5.3	20.8	122.0

MIT, Massachusetts Institute of Technology; AAD, Aalborg University; AUTH, Aristotle University of Thessaloniki; UHA, University of Haute Alsace; DLUT, Dalian University of Technology; SUA, Shiraz University

Step 1: Due to the original databases collected by each institution, multiple sets of heart sound records may have been obtained from the same subjects. To ensure subject independence, the experiment combined the data from 5 medical institutions (*Dataset*_{*b* − *f*}_) as the training set, while the database *Dataset_a_* was designated separately as the public test set. Additionally, we implemented a downsampling strategy using the *RandomUnderSampler* function in Python to address the data imbalance problem. After balancing the samples, there are 665 positive samples and 665 negative samples. The training set to test set ratio is approximately 7:3. The validation set is derived from the officially provided “validation” dataset, comprising 150 positive and 150 negative samples, each.

Step 2: Further selecting the subset of features that have the most impact on the model benefits resource-constrained federated clients, as it is expected to improve model performance while reducing feature dimensionality. As the FL model in this paper is a novel privacy-preserving gradient tree boosting framework, it conducts FL by constructing boosting trees across multiple federated parties. Using the 6,373-dimensional ComParE feature set, we apply 5 tree-based feature importance analysis methods: *gain*, *total_gain*, *cover*, *total_cover*, and *weight*, along with a *SHAP*-based method to assess their individual contributions to the model. Subsequently, the selected 165 features will be used in the hyperparameter experiments of this study.

Step 3: Since accurate labels exist for all samples in the dataset, to assess the effectiveness of the semisupervised FL algorithm, we introduce the assumption that labels for some samples are absent. Following the *PU* scenario, we designate all negative samples as unlabeled, while also masking a portion of the positive samples as unlabeled. This approach, inspired by a previous study [39], involves randomly selecting 20% of the positive data as labeled positive examples, treating the rest of the data as unlabeled examples. The mask strategy is visually depicted in Fig. 4A, where the unmasked part represents positive samples, and the masked part is unlabeled. This masking strategy is applied to both the training and testing datasets.

Step 4: Following the completion of step 3, we vertically partition the preprocessed dataset, gearing up for the vertical-*SecureBoost* model with *PU* learning. In vertical-FL, datasets across institutions share the same sample space but exhibit different feature spaces. To adhere to this condition, vertical partitioning in this study involves vertically dividing the dataset. Let us consider a dataset *D* = (*X*, *Y*) consisting of a feature set *X* and a label set *Y*, partitioned into *guest* = (*X*_1_, *Y*) and *host* = (*X*_2_), where *guest* represents the federated participant with labels, *host* denotes the unlabeled participant, and *X* = *X*_1_ ∪ *X*_2_. The classifier’s objective is to label the unlabeled samples within the masked segment and accurately classify the unmasked positive samples.

### Experimental design

#### Vertically federated XGBoost (vertical-*SecureBoost*)

FL is an emerging machine learning paradigm that leverages decentralized data and distributed learning. It offers a novel solution for collaborative modeling across multiple healthcare institutions. In the traditional horizontal-FL approach, participating institutions initially train their models using local data. Subsequently, they transfer the parameters of these local models, such as the gradients of neural networks, to a central server for aggregation. This process enables the construction of robust global models without sharing raw data. Horizontal-FL requires alignment of feature spaces among participants, which is an ideal scenario. This paper considers medical institutions as federated participants and studies the same patient population with different medical record feature spaces, which is consistent with the vertical-FL scenario. Vertical FL, also known as feature-partitioned FL, is suitable for scenarios where medical institutions share the same patient population. In other words, the data of these institutions have the same sample space but different feature spaces.

 In this study, we employ a vertical-FL model named vertical-*SecureBoost* for semisupervised FL learning. In the vertical-*SecureBoost* setting, only one client has labels, while other clients only have features. The client with labels is referred to as the *guest* party, and the others are termed *host* parties. The role of the *guest* party is analogous to the central server in horizontal-FL. In real medical scenarios, some FL participants have unlabeled data and only serve as feature providers. In response, the semisupervised FL of this paper aims to address the problem of missing and unlabeled labels in federated medical institutions.

**Fig. 2.**
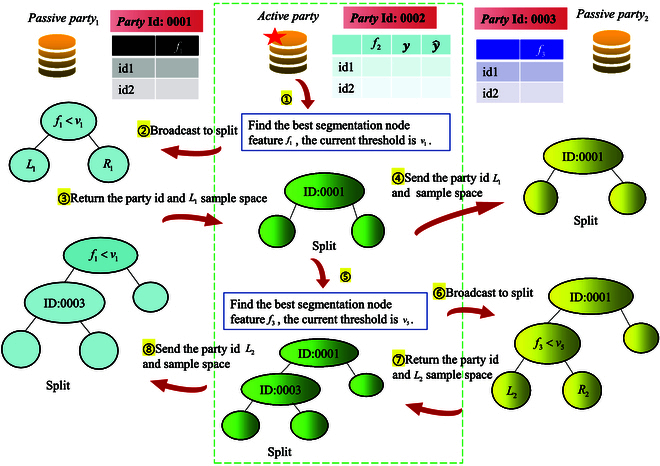
The splitting mechanism for privacy preservation. Vertical-*SecureBoost* guarantees the privacy and security in the process when multiple parties jointly build the tree model. When the *guest* party figures out the best split feature, it will notify the party that holds the feature, denoted as *host*. Then, the *host* will search for its threshold value, split the local model, and get the left children and right children. After splitting the local model, the *host* will transfer its party id and the sample space in the left children node to the *guest* party, since the sample space in the right children can be inferred from the left children. The *guest* party then records the party id in the current node and splits the local tree model. Then, the *guest* party will send party id and the sample space in the left children node to the remaining party. In this way, although all the parties share the same tree model, the recorded information of each node of each party’s tree model may be different. Each party can only have the authority to see its own data information.

The *guest* party, holding the class labels, is responsible for computing gradient values for all samples and transmitting them to all *host* parties. Additionally, the *guest* party is tasked with aggregating feature bins from *host* parties, decrypting gradient histograms, traversing them, and determining the optimal split point along with the corresponding feature. For *host* parties, the main function is to compute their own feature bins and local gradient histograms based on the encrypted gradient values of all samples transmitted by the *guest* party. Upon receiving the broadcast from the *guest* party regarding the optimal splitting feature, the *host* party holding that feature must determine the corresponding threshold value. The node-splitting mechanism of the tree model in vertical-*SecureBoost* is illustrated in Fig. 2.

#### *PU* classification scenario

*PU* classification is prevalent in real-world applications such as healthcare and bioinformatics. The data consist of an incomplete set of positive samples and a set of unlabeled samples that may be either positive or negative.

Stated formally, let  *y*∈ {0, 1} be a binary label, *x* be the feature matrix, *s* = 1 if the sample is labeled, and *s* = 0 if the sample is not labeled. If *y* = 1, then *s* = 1. But if *s* = 0, *y* can be either 1 or 0. So, we have *p*(*s* = 1| *x*, *y* = 0) = 0, which means that the probability that a negative sample *x* appears in the labeled set is zero.

#### Theoretical basis of the naive *PU* training strategy

In this study, we adopt a naive *PU* training strategy, modeling only from positive and unlabeled data. This strategy initially treats all unlabeled samples as negative sample and then trains the model accordingly. High-scoring initial samples are identified as positive label, while the rest are labeled negative. Subsequently, the second classifier is trained. This process is repeated until the unlabeled samples yield the desired result.

The naive *PU* training strategy has been proved to be reasonable by the work [39]. It shows that a classifier trained on positive and unlabeled examples predicts probabilities that differ by only a constant factor from the true conditional probabilities of being positive. Let *f*(*x*) = *p*(*y* = 1| *x*), *g*(*x*) = *p*(*s* = 1| *x*). *f* is a traditional probabilistic classifier, while *g* is a nontraditional one. It can be proved that *p*(*y* = 1| *x*) = *p*(*s* = 1| *x*)/*c*, where *c* is a constant. The proof is that *p*(*s* = 1| *x*) = *p*(*y* = 1, *s* = 1| *x*) = *p*(*y* = 1| *x*)*p*(*s* = 1| *y* = 1, *x*) = *p*(*y* = 1| *x*)*p*(*s* = 1| *y* = 1); according to the definition of the *PU* scenario, *p*(*s* = 1| *y* = 1) is a constant. It can be noticed that *f* is an increasing function of *g*. This means that if the classifier *f* is only used to rank examples *x* according to the chance that they belong to class *y* = 1, then classifier *g* can be used directly instead of *f*, which verifies the rationality of the naive *PU* training strategy. The description of relevant variables is shown in Table 2.

**Table 2.** List of notations used in the semisupervised vertical-*SecureBoost* model

List of notations	Explanations
*P*	Dataset with positive labels
*U*	Unlabeled dataset
*X*_1_ ∈ *R^n×a^*	Feature matrix of the labeled dataset for the *guest* party
*X*_2_ ∈ *R^m×a^*	Feature matrix of the unlabeled dataset for the *guest* party
*X*_3_ ∈ *R*^(*n+m*)×*b*^	Feature matrix of the *host* party
*s* ∈ {0,1}	*s* = 1 means the sample is labeled, *s* = 0 means the sample is unlabeled.
*y*	True labels of the samples
*ŷ*	The predicted label of the sample in the previous round
*i*	Sample identification number
*j*	Client identification number
*k*	Feature identification number
*q*	Feature bin identification number
*d_j_*	Number of features for the *j*th client
*u* *_kq_*	The splitting value of the *q*th feature bin for the *k*th feature
*g_i_*	The first-order derivative of the loss function with respect to the predicted labels of the previous round, denoted as $g_{i}=\frac{\partial l\left(y_{i}{\hat{y}}_{i}^{\;t–1}\right)}{\partial {\hat{y}}_{i}^{\;t–1}}$
*h_i_*	The second-order derivative of the loss function with respect to the predicted labels of the previous round, denoted as $h_{i}=\frac{\partial^{2}l\left(y_{i}{\hat{y}}_{i}^{\;t-1}\right)}{\partial^{2}{\hat{y}}_{i}^{\;t-1}}$

#### Workflow of *PU* vertical-*SecureBoost*

*PU* is applicable to classification tasks in the vertical-FL scenario. The constructed semisupervised FL model can be trained using positive samples and unlabeled samples, and the prediction of unlabeled samples is completed based on the trained model. As the labels change, the data distribution also undergoes alterations, requiring the model to rely on the updated data for continued training. The iterative process continues for multiple rounds until the labels in the dataset converge under predefined rules. Due to the absence of overlapping users among medical institutions, we merged data from five institutions for building the vertical-FL model. Specifically, the multi-dimensional table data extracted after merging are partitioned into 2 segments based on the feature columns, representing the feature spaces for the federated participants—*guest* and *host*, respectively. The FL participants, *guest* and *host*, meet the requirement that the sample space is the same but the feature space is different, thus enabling vertical-FL modeling. In this study, we designate the medical institution data warehouse as the federated client and establish 2 federated parties for vertical-FL modeling: the *guest* party and the *host* party.

**Fig. 3.**
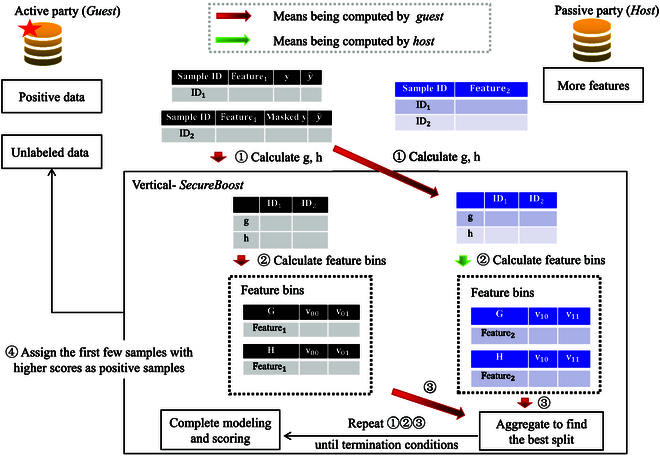
Rough outline of the workflow of semisupervised vertical-*SecureBoost*. The illustration shows 2 federation participants, a *host* party and a *guest* party. In the guest side, *ID*_1_ represents labeled samples, *ID*_2_ represents unlabeled samples. Masked *y* refers to our treatment of unlabeled samples based on the *PU* learning strategy. *y* represents the predictions of the samples from the previous round. The host side does not have labels and only provides features. In stage 1, the *guest* side calculates the first-order derivative (*g_i_*) and the second-order derivative (*h_i_*) of the loss function for each sample *ID* based on the real or masked labels and the predictions from the previous round, and sends this information to the *host* side. In stage 2, all parties calculate feature bins based on the information from *g_i_* and *h_i_*, and this relevant information is transmitted to the *guest* side. In stage 3, the guest side aggregates all the feature bin information from the participating parties and iteratively calculates the best split points for the tree. In stage 4, the algorithm ranks the samples based on the scoring values obtained using the *PU* learning strategy.

**Fig. 4.**
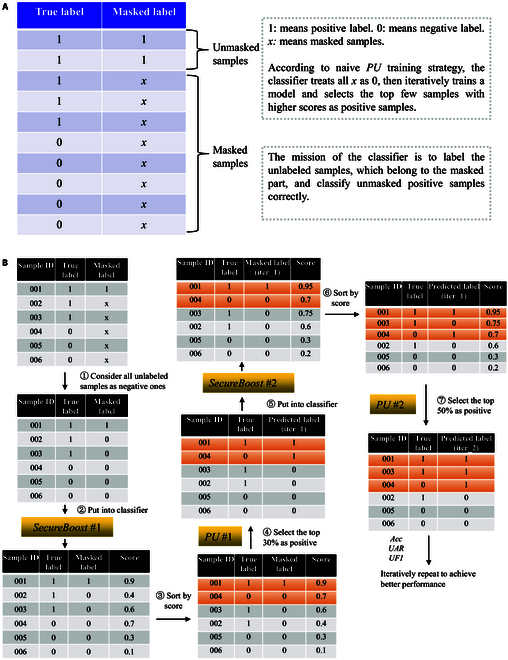
Rough outline of the workflow of semisupervised vertical-*SecureBoost*. (A) Mask strategy on the dataset. (B) Simple concrete example of the training process.

Figure 3 illustrates the workflow of semisupervised vertical-*SecureBoost* with a naive *PU* training strategy, providing additional details on each component. As the *guest* participant in the FL, the *guest* holds 2 types of data: positive samples and unlabeled samples. In the data preprocessing stage, unlabeled samples are treated as negative samples, and the process incorporates the vertical-*SecureBoost* FL algorithm. The trained federated model is used to predict the unlabeled intersection data of the *guest* participant. Subsequently, these data are sorted based on their predicted probabilities, and those exceeding a predefined threshold are selected. Positive labels are then assigned to these selected high-probability unlabeled intersection data. Figure 4A illustrates the masking strategy used in our experiments with the selected dataset. Figure 4B provides a simple concrete example to illustrate the training process. Algorithm 1 describes the pseudocodes detailing the basic principles and workflow of semisupervised vertical-*SecureBoost* with a naive *PU* training strategy.

#### Evaluation metrics

The multi-institutional heart sound database reflects imbalances in sample size and class distribution across institutions. This study uses the following evaluation metrics, in addition to traditional methods such as accuracy (*Acc*), to measure model performance. Given are *C* classes, true positives (*TP*), false negatives (*FN*), false positives (*FP*), and true negatives (*TN*).

We utilize the unweighted average recall (*UAR*) and the unweighted *F1-Score* (*UF1*) to evaluate the performance of the diagnostic model. The importance of the *UAR* metric lies in its ability to give equal importance to the performance of each class. Therefore, *UAR* is especially valuable for evaluating models on datasets where some classes are under-represented. *UAR* is calculated as:

$$\mathrm{UA}R_{c}=\frac{\sum_{i=1}^{C}‍\text{Recal}l_{i}}{C}$$ (1)

and *UF1* can be formulated as:

$$\mathrm{UF}1_{c}=\frac{2*TP_{C}}{2*TP_{C}+FP_{C}+FN_{C}}$$ (2)

## Results

Our main objective is to investigate the hyperparameter configurations of the vertical-*SecureBoost* model with native *PU* learning. Subsequently, we will conduct comparative experiments to assess model performance using various feature importance analysis methods, aiming to provide valuable insights into abnormal heart sound detection. The experiment includes essential parameters within the interactive learning processes of both the *SecureBoost* and *PU* components, along with the selection of 165 features determined by feature importance analysis methods.

### Model hyperparameter experiment

We explore crucial hyperparameter settings in semisupervised FL models through 2 sets of experiments. The first set involves configuring the proportion parameter in the *PU* component and determining the number of trees in the *SecureBoost* component. The second set focuses on establishing the optimal numbers of *SecureBoost* and *PU* components in semisupervised FL. The “proportion in *PU*” refers to the percentage of top samples considered as positive when executing the current *PU*, determined based on the sorted scores of samples labeled by the preceding *SecureBoost* classifier.

#### Relationship between the first *PU (PU*_1_*)* proportion and model performance

The *PU* learning strategy enables the FL model to directly learn from a limited set of positive samples and a large pool of unlabeled samples. A control group experiment is conducted to analyze the impact of different proportions in *PU*_1_ on FL global model performance while keeping other settings fixed. Since the preprocessed data class is balanced with equal proportion of positive and negative samples, we set the final *PU* (*PU*_2_) proportion to 0.5. This helps the model’s predictions for the samples converge to an equal distribution of positive and negative outcomes. As depicted in Table 3, the model’s performance improves with increasing proportions of *PU*_1_. However, when the proportion exceeds 30%, the model metrics start to decline. The model achieves optimal values (*Acc*: 84.36%, *UAR*: 84.33%, *UF1*: 84.35%) when the proportion in *PU*_1_ is 30%.

**Table 3.** Mean testing performance (in [%]) of 50 repetitions of the semisupervised FL model. Exploring the relationship between the proportion in the first *PU* (*PU*_1_) and model performance. Fixed parameters: The proportion in the second *PU* (*PU*_2_) is 0.5. The number of trees in *SecureBoost*_{1, 2, 3}_ is 10, 20, and 30, respectively, and the depth of the trees is 3.

*PU*_1_ proportion	*PU*_2_ proportion	*Acc*	*UAR*	*UF1*
0.1	0.5	80.601	80.687	80.600
0.2	0.5	81.353	81.411	81.350
0.3	0.5	84.360	84.338	84.351
0.35	0.5	82.105	82.108	82.096
0.4	0.5	82.105	82.136	82.099
0.5	0.5	82.105	82.136	82.099

Another control group experiment involves varying the number of tree models in the *SecureBoost* component. This pertains to the impact of the *SecureBoost* model complexity on the performance of the semisupervised FL model. The experiment fixed 3 *SecureBoost* components, each with relevant parameters, and examined the performance variation of the FL model with 10, 20, 30, and 40 trees within each component. As indicated in Table 4, the semisupervised FL model achieved its best performance (*Acc*: 84.36%, *UAR*: 84.33%, *UF1*: 84.35%) when *SecureBoost*_1_ has 10 trees, *SecureBoost*_2_ has 20 trees, and *SecureBoost*_3_ has 30 trees.

**Table 4.** Mean testing performance (in [%]) of 50 repetitions of the semisupervised FL model. Exploring the impact of the number of tree models in the *SecureBoost* component on the performance of semisupervised FL. *Fixed parameters:* proportion 0.3 in the first *PU* (*PU*_1_), proportion 0.5 in the second *PU* (*PU*_2_). The depth of the tree in *SecureBoost* is 3.

FL components	Number of trees	*Acc*	*UAR*	*UF1*
*SecureBoost*_{1, 2, 3}_	10, 10, 10	80.977	80.936	80.959
*SecureBoost*_{1, 2, 3}_	20, 20, 20	81.353	81.326	81.338
*SecureBoost*_{1, 2, 3}_	30, 30, 30	82.105	82.164	82.102
*SecureBoost*_{1, 2, 3}_	40, 40, 40	81.062	81.034	81.058
*SecureBoost*_{1, 2, 3}_	10, 20, 30	84.360	84.338	84.351

#### Relationship between the number of *PU* and model performance

Figure 4 illustrates the interactive learning process between the *SecureBoost* and *PU* components in the FL model based on *PU*. The number of *SecureBoost* and *PU* components determines the iterations or rounds of the learning process. In the control experiment, we varied the number of *PUs* from 1 to 3, and the corresponding number of *SecureBoost* components from 2 to 4. As indicated in Table 5, the semisupervised FL model achieves its optimal performance with 2 *PU* components and 3 *SecureBoost* components. Experimental results, in conjunction with tree models, demonstrate that we can achieve higher classification performance of the semisupervised FL model with relatively lower model complexity.

**Table 5.** Mean testing performance (in [%]) of 50 repetitions of the semisupervised FL model. Exploring the impact of the number of *SecureBoost* and *PU* components on model performance. Fixed parameters: The proportion for *PU*_1_ is 0.3, and for *PU*_2_, it is 0.5. The number of trees in *SecureBoost*_{1, 2, 3}_ is 10, 20, and 30 respectively, and the depth of the trees is 3.

Number of *PU*	Number of *SecureBoost*	*Acc*	*UAR*	*UF1*
1 (*PU*_{1}_)	2 (*SecureBoost*_{1, 2}_)	80.225	80.240	80.216
2 (*PU*_{1, 2}_)	3 (*SecureBoost*_{1, 2, 3}_)	80.601	80.630	80.594
3 (*PU*_{1, 2, 3}_)	4 (*SecureBoost*_{1, 2, 3, 4}_)	80.601	80.622	80.590


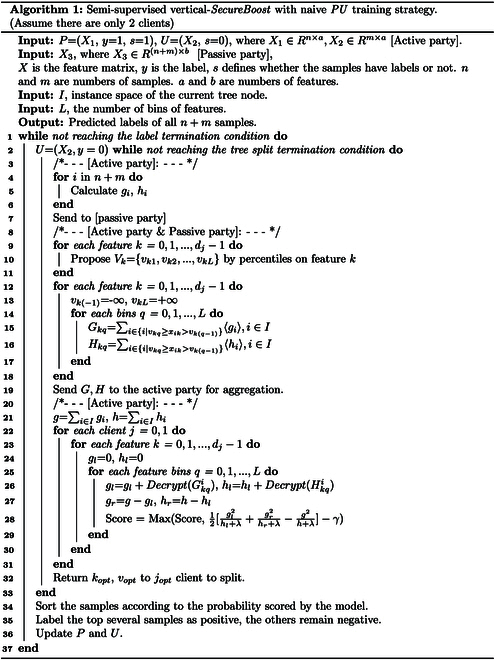


### Comparative experiment on feature selection methods

This study has 2 main objectives for the semisupervised FL classification model. First, it should perform well, accurately predicting the output of given input features. Second, the model should be interpretable, providing an understanding of the relationship between input features and output. This is crucial when using auxiliary diagnostic models in the sensitive field of healthcare. For instance, in a cardiac auscultation model, it is vital to predict the patient’s diagnosis and understand which features contribute to the result.

Feature importance analysis is a widely used method for interpreting classification models. It quantifies the individual contributions of specific features to a given classifier. Thus, the importance of input data features is model-dependent. In this study, we compared the effects of various feature importance analysis methods on the classification performance of our model, utilizing a high-dimensional feature set extracted from the original heart sound recordings.

In the vertical-FL framework, each federated participant has a distinct feature space. Furthermore, we aim to identify which features contribute most to the performance of the semisupervised FL model in this study. Since vertical-*SecureBoost* is implemented based on the XGBoost model, we employed 5 tree-based feature selection methods: *gain*, *total_gain*, *cover*, *total_cover*, and *weight*. Additionally, we conducted comparative experiments using the *SHAP* method. Although they are technically related and partially overlap, there is a distinction between feature importance and feature selection. The experiments show that these methods consistently filter the same set of 165 contributing heart sound features (including LLD features and statistical features), with only differences in the importance ranking of these features. The table in the Appendix presents the computed results (feature coefficients and importance values) for the 165 features, sorted by feature importance from the *SHAP* method. In the comparative experiments, selecting the top 165 features based on the *SHAP* method yielded optimal model performance (*Acc*: 84.36%, *UAR*: 84.33%, *UF1*: 84.35%). The model results for other feature selection methods under the same conditions are compared in Table 6. Moreover, our optimal model performance closely matches that of the supervised *SecureBoost* model when using 30 trees and a tree depth of 3. Comparative experimental demonstrate that the semisupervised *SecureBoost* model efficiently identifies the heart sound features that contribute most, particularly when employing the *SHAP* feature importance analysis method. The advantage lies in selecting fewer features to achieve superior classification performance, providing clear benefits over other methods. To further demonstrate the superiority of the proposed method, we conduct a comparison with the semisupervised FL algorithms. FedPU (https://github.com/littleSunlxy/FedPU-torch) [34] and FedMatch (https://github.com/wyjeong/FedMatch) [35], compared with the optimization models in this paper, represent the most comparable and state-of-the-art semisupervised FL models. Table 6 presents the performance comparison among FedPU, FedMatch, and the proposed method. Given the limited data resources in this study, the proposed method achieves state-of-the-art performance on the multi-institutional heart sound database. This also demonstrates that our method outperforms other semisupervised FL methods under low-resource conditions.

**Table 6.** Mean testing performance (in [%]) of 50 repetitions of semisupervised and supervised FL models. Performance comparison of semisupervised FL models when utilizing different feature importance analysis methods. Fixed parameters: In semisupervised learning, the proportion for *PU*_1_ is 0.3, and for *PU*_2_, it is 0.5. The number of trees in *SecureBoost*_{1, 2, 3}_ is 10, 20, and 30, respectively. In supervised learning, the number of trees is 30 and the depth of the trees is 3.

Methods	*Acc*	*UAR*	*UF1*
*SecureBoost* (Supervised)	84.628	84.971	85.107
*FedPU* [34](*SHAP* feature set)	75.103	68.330	68.001
*FedMatch* [35] (*SHAP* feature set)	65.007	65.791	62.205
*SecureBoost* with naive *PU*	-	-	-
*gain*	81.353	81.383	81.347
*total_gain*	80.977	80.964	80.964
*cover*	79.473	79.600	79.473
*total_cover*	81.353	81.383	81.347
*weight*	80.601	80.743	80.601
*SHAP*	84.360	84.338	84.351

## Discussion

We will now discuss 3 aspects: the application of the semisupervised FL model in heart sound classification, the identification of the most important features, and whether the crucial features vary depending on the technique used.

Application of the semisupervised FL model. We study the problem of learning from positive and unlabeled (*PU*) data in the federated setting. Specifically, we concentrate on scenarios where some clients have only positive and unlabeled samples, while others have only unlabeled samples. The semisupervised FL model can effectively learn from different institutions with a limited pool of positive samples and unlabeled samples. We validated the effectiveness of this framework on real-world heart sound recordings through a series of experiments. Additionally, this framework demonstrates the ability to achieve better classification performance with relatively low model complexity. When utilizing the *SHAP* feature importance analysis method, all metrics consistently reach above 84%. The semisupervised FL model can conduct multi-institutional federated modeling without sharing local medical institution data. This helps address the issue of medical data silos and partially safeguards patient privacy. However, it is worth noting that the limited data and the relatively simple *PU* strategy mean that the performance of the FL model in medical diagnosis needs to be improved. To this end, we are collaborating with multiple medical institutions to build a larger, high-quality multi-institutional heart sound database, such as https://www.vob-bit.org, as part of our current work. In practical applications, assessing the performance of the proposed model necessitates considering the diverse environments of each medical institution. Future work should explore various factors in practical applications, such as the number of federated participants, communication costs, data distribution, and FL modeling based on multimodal data [40,41].

What features are the most important? To refine effective representations of heart sounds from the 6,373 features in the ComParE feature set for the model, we employed various feature importance methods. Consistently, these methods identified the same 165 features contributing to the model, albeit with differences in importance ranking. The key statistical findings are as follows: The most influential features encompass 73 related “*udSpec*” features, with 57 related to “*udSpec*_*Rfilt*” and 6 related to “*udspecRasta*.” Additionally, there are 45 features associated with “*fcc*_*sma*^”^ and 36 features linked to “*cm*_*fftMag*,” including 30 features tied to “*cm*_*fftMag*_*spectral*” and 5 features associated with “*cm*_*RMSenergy*.” This implies that distinct methods can identify the same effective features for the same classification model. Furthermore, the features extracted from the heart sound data exhibit high correlation, making the classification task straightforward. Thus, different feature importance analysis methods can enable the FL model to achieve better classification accuracy.

Do the most important features differ depending on the technique? The most important features indeed depend on the method used. Our experiments indicate that the *SHAP* method provides better results, as the model’s performance is optimal and stable when the first 165 *SHAP* features are selected. By selecting fewer features and achieving optimal performance in the analyzed cases, *SHAP* has a clear advantage over other methods. Ultimately, this study provides insights into screening one-dimensional acoustic signal features for abnormal heart sound examination. It is noteworthy that this framework, rooted in traditional machine learning, is designed for processing one-dimensional tabular data rather than phonocardiogram (PCG) images. Although model interpretability was not the primary focus, the feature importance analysis in this paper lays the foundation for future FL research on feature-based interpretability.

### Conclusion

This study was motivated by 2 primary objectives. First, we assessed the classification performance of the semisupervised FL model using real-world heart sound recordings. Second, we investigated the influence of various feature importance methods on the model’s classification performance. Utilizing the classical ComParE feature set, we identified 165 features contributing to the model. Notably, we observed superior performance in heart sound classification with the *SHAP*-based method, which selected fewer features in the analyzed cases while meeting the model’s performance criteria.

The framework employed a naive *PU* learning strategy, one of the most basic semisupervised learning methods. In future work, we will explore more complex *PU* training strategies to enhance the performance of the FL model. Moreover, we intend to replicate the proposed analytical scheme on a larger scale, particularly aiming to implement the techniques utilized in neural network-based FL frameworks. The synergy of advanced nonlinear FL models and sophisticated *PU* learning strategies is expected to demonstrate significant potential for extensive PCG signals.

## Appendix

**Table A1.** Based on the ComParE feature set, we present the selected 165 heart sound features and their corresponding computational results

Feature name	SHAP value	Weight	Gain	Cover	Total gain	Total cover
udspecRasta_lengthL1norm_sma_de_stddevRisingSlope numeric	0.1749088	4	59.07317352	1330	236.2926941	5320
fcc_sma[5]_peakMeanRel numeric	0.066608705	2	33.78628922	908	67.57257843	1816
fcc_sma[4]_percentile99.0 numeric	0.027021766	1	9.735995293	1330	9.735995293	1330
cm_fftMag_spectralSkewness_sma_meanFallingSlope numeric	0.01917158	1	5.615310669	1330	5.615310669	1330
cm_fftMag_spectralSlope_sma_risetime numeric	0.016092975	1	3.445723057	1330	3.445723057	1330
udSpec_Rfilt_sma_de[22]_quartile3 numeric	0.015471268	2	6.0129776	374	12.0259552	748
cm_fftMag_spectralFlux_sma_de_quartile2 numeric	0.014841603	1	6.25514555	747	6.25514555	747
udspec_lengthL1norm_sma_de_lpc0 numeric	0.014638417	1	13.98928738	328	13.98928738	328
udSpec_Rfilt_sma[11]_risetime numeric	0.014299023	3	1.715965867	611	5.14789772	1833
udSpec_Rfilt_sma_de[23]_quartile3 numeric	0.014214776	1	9.673725128	679	9.673725128	679
fcc_sma[4]_iqr1-3 numeric	0.013853458	2	7.019974709	430	14.03994942	860
oicingFinalUnclipped_sma_flatness numeric	0.013512484	1	8.57629776	498	8.57629776	498
udSpec_Rfilt_sma[0]_quartile2 numeric	0.013094406	1	2.308807135	1330	2.308807135	1330
oicingFinalUnclipped_sma_lpc0 numeric	0.01280389	1	7.025602341	596	7.025602341	596
cm_fftMag_spectralHarmonicity_sma_percentile1.0 numeric	0.012099205	1	11.92963409	258	11.92963409	258
cm_fftMag_spectralCentroid_sma_skewness numeric	0.011240978	2	0.81099081	942	1.621981621	1884
fcc_sma[3]_peakMeanAbs numeric	0.011181817	1	14.97934723	451	14.97934723	451
udSpec_Rfilt_sma_de[13]_stddevRisingSlope numeric	0.010769798	1	1.756378412	1330	1.756378412	1330
fcc_sma[3]_iqr2-3 numeric	0.010016562	1	5.267727852	738	5.267727852	738
cm_fftMag_spectralSkewness_sma_de_lpgain numeric	0.009126852	1	1.210110903	1330	1.210110903	1330
oicingFinalUnclipped_sma_lpgain numeric	0.007829903	1	2.564095974	611	2.564095974	611
udSpec_Rfilt_sma_de[2]_risetime numeric	0.007798587	1	1.246006727	1330	1.246006727	1330
cm_RMSenergy_sma_peakRangeAbs numeric	0.007496908	1	7.285607815	734	7.285607815	734
cm_fftMag_spectralCentroid_sma_minRangeRel numeric	0.007475868	1	0.660296619	1330	0.660296619	1330
cm_fftMag_spectralVariance_sma_flatness numeric	0.007420569	1	2.622623205	775	2.622623205	775
fcc_sma[3]_amean numeric	0.006865831	1	3.782421112	393	3.782421112	393
udSpec_Rfilt_sma[2]_linregerrQ numeric	0.006835031	1	0.912325859	1326	0.912325859	1326
cm_RMSenergy_sma_flatness numeric	0.006573637	1	6.422821045	749	6.422821045	749
udSpec_Rfilt_sma[5]_quartile3 numeric	0.006294543	1	0.478050798	1321	0.478050798	1321
udSpec_Rfilt_sma[5]_iqr1-2 numeric	0.005954946	1	1.598445177	1111	1.598445177	1111
fcc_sma[12]_iqr2-3 numeric	0.005753407	1	6.725850105	583	6.725850105	583
fcc_sma[13]_lpgain numeric	0.005692956	1	0.502746999	1298	0.502746999	1298
cm_fftMag_fband250-650_sma_de_peakDistStddev numeric	0.005576141	1	0.389642864	1330	0.389642864	1330
udSpec_Rfilt_sma_de[24]_quartile2 numeric	0.005573068	2	1.020026922	1330	2.040053844	2660
udSpec_Rfilt_sma[6]_quartile3 numeric	0.005310398	1	5.443786621	434	5.443786621	434
udspecRasta_lengthL1norm_sma_de_iqr1-2 numeric	0.005278943	1	1.099442482	1018	1.099442482	1018
fcc_sma[5]_lpgain numeric	0.005124319	1	1.139160156	424	1.139160156	424
udSpec_Rfilt_sma_de[13]_meanRisingSlope numeric	0.005056385	1	0.394382507	1280	0.394382507	1280
fcc_sma_de[3]_kurtosis numeric	0.004974036	1	2.690096855	819	2.690096855	819
fcc_sma_de[2]_percentile1.0 numeric	0.00495234	1	0.621264398	790	0.621264398	790
cm_fftMag_fband250-650_sma_linregc1 numeric	0.004844865	1	0.281745851	1312	0.281745851	1312
fcc_sma_de[2]_skewness numeric	0.004828318	1	0.945549786	770	0.945549786	770
udspec_lengthL1norm_sma_meanSegLen numeric	0.004793007	1	1.342338324	979	1.342338324	979
udSpec_Rfilt_sma[6]_meanSegLen numeric	0.004744658	1	0.997637093	1169	0.997637093	1169
udSpec_Rfilt_sma[0]_risetime numeric	0.004732204	1	3.225561857	74	3.225561857	74
fcc_sma[2]_maxSegLen numeric	0.004728207	1	0.519239247	1327	0.519239247	1327
udSpec_Rfilt_sma_de[14]_stddevRisingSlope numeric	0.00470226	1	1.475333691	391	1.475333691	391
fcc_sma_de[2]_quartile2 numeric	0.004379132	1	1.170669794	1303	1.170669794	1303
fcc_sma_de[11]_peakDistStddev numeric	0.004351366	1	0.770152211	1330	0.770152211	1330
fcc_sma_de[9]_peakDistStddev numeric	0.004338105	1	0.388319731	1325	0.388319731	1325
cm_fftMag_spectralVariance_sma_linregc2 numeric	0.004324389	1	2.174813986	879	2.174813986	879
fcc_sma[1]_quartile1 numeric	0.004088204	2	2.74508667	351.5	5.49017334	703
udSpec_Rfilt_sma[6]_iqr2-3 numeric	0.004022839	1	1.191879869	1294	1.191879869	1294
cm_fftMag_psySharpness_sma_minRangeRel numeric	0.003896863	1	0.401606768	1062	0.401606768	1062
udspecRasta_lengthL1norm_sma_meanSegLen numeric	0.003853667	1	0.445445478	1295	0.445445478	1295
udSpec_Rfilt_sma_de[3]_leftctime numeric	0.003800792	1	2.882632017	592	2.882632017	592
fcc_sma[10]_peakRangeRel numeric	0.003772016	2	1.125457048	1041.5	2.250914097	2083
fcc_sma_de[5]_lpc1 numeric	0.003659269	1	2.361129761	923	2.361129761	923
cm_fftMag_spectralSkewness_sma_lpc0 numeric	0.003499466	1	3.718276978	523	3.718276978	523
udSpec_Rfilt_sma_de[12]_stddevFallingSlope numeric	0.003484988	1	1.881630421	407	1.881630421	407
fcc_sma[2]_upleveltime50 numeric	0.003321341	1	0.834810019	983	0.834810019	983
cm_fftMag_spectralSlope_sma_de_quartile3 numeric	0.003269716	1	2.736748695	569	2.736748695	569
fcc_sma_de[4]_pctlrange0-1 numeric	0.003264664	1	1.344154358	555	1.344154358	555
udSpec_Rfilt_sma[10]_meanSegLen numeric	0.003213854	1	0.999613822	1074	0.999613822	1074
cm_fftMag_spectralSkewness_sma_de_flatness numeric	0.003093224	1	3.515030384	127	3.515030384	127
cm_fftMag_spectralSkewness_sma_de_percentile99.0 numeric	0.00309266	1	1.360512137	950	1.360512137	950
udSpec_Rfilt_sma[10]_lpc1 numeric	0.003090039	1	0.669033051	888	0.669033051	888
fcc_sma[7]_linregerrQ numeric	0.003059623	1	0.362798691	1302	0.362798691	1302
fcc_sma[13]_iqr2-3 numeric	0.00305221	1	2.921410799	89	2.921410799	89
fcc_sma_de[9]_quartile2 numeric	0.002958984	1	0.74704951	901	0.74704951	901
fcc_sma[8]_range numeric	0.002835295	1	0.767194033	1327	0.767194033	1327
fcc_sma_de[13]_risetime numeric	0.002822805	1	1.911473036	60	1.911473036	60
cm_fftMag_spectralSlope_sma_linregc1 numeric	0.002801212	1	1.542387009	347	1.542387009	347
udSpec_Rfilt_sma[11]_segLenStddev numeric	0.002742412	1	0.933002472	385	0.933002472	385
fcc_sma_de[7]_pctlrange0-1 numeric	0.002739604	1	1.344755292	506	1.344755292	506
udSpec_Rfilt_sma_de[6]_percentile1.0 numeric	0.002661523	1	1.024646759	1330	1.024646759	1330
udSpec_Rfilt_sma[15]_peakRangeRel numeric	0.001648004	1	0.53542912	263	0.53542912	263
fcc_sma[2]_linregc1 numeric	0.001599217	1	0.394384265	600	0.394384265	600
cm_fftMag_psySharpness_sma_linregc1 numeric	0.001595959	1	0.334819168	1324	0.334819168	1324
udSpec_Rfilt_sma[7]_leftctime numeric	0.001591305	1	1.373440266	44	1.373440266	44
fcc_sma[3]_upleveltime90 numeric	0.001587785	1	2.038755417	760	2.038755417	760
fcc_sma[5]_rqmean numeric	0.001532889	2	0.726613462	1266.5	1.453226924	2533
fcc_sma[10]_skewness numeric	0.001477398	1	0.527558625	324	0.527558625	324
cm_fftMag_spectralKurtosis_sma_de_flatness numeric	0.001469919	1	0.467760682	1241	0.467760682	1241
udSpec_Rfilt_sma[25]_upleveltime50 numeric	0.001460258	1	0.302553505	1326	0.302553505	1326
fcc_sma_de[5]_lpc4 numeric	0.001403587	1	0.539424777	1291	0.539424777	1291
udSpec_Rfilt_sma_de[10]_upleveltime90 numeric	0.001380694	1	1.193989992	23	1.193989992	23
cm_fftMag_spectralRollOff75.0_sma_de_stddevFallingSlope numeric	0.001334538	1	0.547998667	798	0.547998667	798
oicingFinalUnclipped_sma_range numeric	0.00132699	1	1.335298538	31	1.335298538	31
udSpec_Rfilt_sma[6]_pctlrange0-1 numeric	0.001314231	1	0.401833385	1185	0.401833385	1185
cm_fftMag_fband250-650_sma_de_range numeric	0.001303879	1	0.647293746	507	0.647293746	507
oicingFinalUnclipped_sma_de_quartile3 numeric	0.001280073	1	0.825291157	49	0.825291157	49
udSpec_Rfilt_sma[17]_lpc3 numeric	0.001216713	1	0.705320001	55	0.705320001	55
fcc_sma[6]_qregc1 numeric	0.001205926	1	0.807898402	100	0.807898402	100
cm_fftMag_fband1000-4000_sma_de_minPos numeric	0.00111533	1	0.484175861	522	0.484175861	522
udSpec_Rfilt_sma_de[25]_minSegLen numeric	0.001072463	1	1.136362314	33	1.136362314	33
udSpec_Rfilt_sma_de[6]_lpc4 numeric	0.001038906	1	0.51261425	532	0.51261425	532
cm_fftMag_spectralSlope_sma_minPos numeric	0.001018498	1	1.17227602	775	1.17227602	775
udSpec_Rfilt_sma_de[22]_skewness numeric	0.000986683	1	0.63786608	796	0.63786608	796
udspec_lengthL1norm_sma_de_pctlrange0-1 numeric	0.00096522	1	0.421422035	44	0.421422035	44
udSpec_Rfilt_sma_de[3]_quartile2 numeric	0.000914101	1	0.789074838	27	0.789074838	27
cm_fftMag_fband1000-4000_sma_qregc3 numeric	0.000913065	1	0.399933308	712	0.399933308	712
udSpec_Rfilt_sma_de[7]_upleveltime75 numeric	0.000890098	1	0.495113492	1330	0.495113492	1330
fcc_sma[12]_stddevFallingSlope numeric	0.000889177	1	0.514449418	41	0.514449418	41
cm_fftMag_spectralFlux_sma_lpc0 numeric	0.000887822	1	0.672494352	83	0.672494352	83
udSpec_Rfilt_sma_de[17]_peakRangeRel numeric	0.000886993	1	0.509827614	34	0.509827614	34
udSpec_Rfilt_sma_de[0]_maxPos numeric	0.000876428	1	0.861174822	16	0.861174822	16
cm_RMSenergy_sma_de_stddevFallingSlope numeric	0.000875804	1	0.281979769	1330	0.281979769	1330
udSpec_Rfilt_sma[9]_percentile1.0 numeric	0.000866589	1	0.848887801	93	0.848887801	93
cm_fftMag_spectralFlux_sma_lpc4 numeric	0.000853217	1	0.259635895	117	0.259635895	117
cm_fftMag_spectralFlux_sma_peakMeanRel numeric	0.000839713	1	0.686322689	433	0.686322689	433
udSpec_Rfilt_sma_de[4]_quartile2 numeric	0.000838053	1	0.496914715	32	0.496914715	32
cm_fftMag_spectralEntropy_sma_peakDistStddev numeric	0.000832948	1	0.411569834	208	0.411569834	208
udSpec_Rfilt_sma_de[2]_quartile2 numeric	0.000829766	1	0.520026982	24	0.520026982	24
udSpec_Rfilt_sma[12]_upleveltime90 numeric	0.000786718	1	0.829185367	27	0.829185367	27
cm_zcr_sma_de_peakRangeRel numeric	0.00076202	1	0.883337021	1330	0.883337021	1330
udspecRasta_lengthL1norm_sma_quartile1 numeric	0.000733587	1	0.555594325	16	0.555594325	16
cm_fftMag_spectralSkewness_sma_qregc3 numeric	0.000690544	1	0.295283973	27	0.295283973	27
udSpec_Rfilt_sma_de[12]_peakRangeRel numeric	0.00067913	1	0.487163782	35	0.487163782	35
udSpec_Rfilt_sma[13]_lpgain numeric	0.00065719	1	0.308226794	1295	0.308226794	1295
cm_fftMag_spectralVariance_sma_range numeric	0.000638415	1	0.73640269	1119	0.73640269	1119
cm_fftMag_spectralKurtosis_sma_peakMeanMeanDist numeric	0.000616125	1	0.413334399	1330	0.413334399	1330
udSpec_Rfilt_sma[19]_minPos numeric	0.0005893	1	0.760017276	1330	0.760017276	1330
udspec_lengthL1norm_sma_de_meanRisingSlope numeric	0.000573997	1	0.358050197	1330	0.358050197	1330
cm_fftMag_spectralKurtosis_sma_linregc1 numeric	0.00056523	1	0.284590483	1330	0.284590483	1330
udspecRasta_lengthL1norm_sma_de_lpc0 numeric	0.000546952	1	0.879844904	12	0.879844904	12
fcc_sma[2]_lpc2 numeric	0.000519563	1	0.439446568	23	0.439446568	23
udSpec_Rfilt_sma[0]_maxPos numeric	0.000489432	1	0.514279604	1330	0.514279604	1330
udspec_lengthL1norm_sma_leftctime numeric	0.000487373	1	0.960110188	23	0.960110188	23
udSpec_Rfilt_sma[8]_minRangeRel numeric	0.000476519	1	0.255015016	26	0.255015016	26
cm_RMSenergy_sma_iqr1-2 numeric	0.000443091	1	0.301709265	1330	0.301709265	1330
cm_RMSenergy_sma_upleveltime90 numeric	0.000413161	1	0.188143015	19	0.188143015	19
cm_fftMag_spectralRollOff75.0_sma_upleveltime75 numeric	0.000366831	1	0.074845433	31	0.074845433	31
udspecRasta_lengthL1norm_sma_maxPos numeric	0.000324066	1	0.175449252	10	0.175449252	10
fcc_sma[6]_minPos numeric	0.000319397	1	0.101224005	18	0.101224005	18
udspec_lengthL1norm_sma_lpc0 numeric	0.000275772	2	0.0727164	6.5	0.1454328	13
udspec_lengthL1norm_sma_percentile99.0 numeric	0.000275314	1	0.221500084	8	0.221500084	8
udSpec_Rfilt_sma[7]_lpc4 numeric	0.000273154	1	0.122567415	14	0.122567415	14
udSpec_Rfilt_sma[4]_lpc0 numeric	0.000215668	1	0.021040797	14	0.021040797	14
udspec_lengthL1norm_sma_de_risetime numeric	0.000187452	1	0.08378467	9	0.08378467	9
udSpec_Rfilt_sma[16]_percentile1.0 numeric	0.000168175	1	0.068053588	9	0.068053588	9
udspec_lengthL1norm_sma_maxPos numeric	9.77E−05	1	0.018804565	8	0.018804565	8
udspec_lengthL1norm_sma_risetime numeric	8.27E−05	1	0.017634902	8	0.017634902	8

## Data Availability

This study utilizes heart sound recordings from the PhysioNet/Computing in Cardiology Challenge, which provides a publicly available heart sound database. The database can be accessed at: https://physionet.org/content/challenge-2016/1.0.0 . Additional preprocessed data relevant to this paper may be requested from the author via email.

## References

[1] Timmis A, Vardas P, Townsend N, Torbica A, Katus H, De Smedt D, Gale CP, Maggioni AP, Petersen SE, Huculeci R, et al. European Society of Cardiology: Cardiovascular disease statistics 2021. Eur Heart J. 2022;43(8):716–799. 35016208 10.1093/eurheartj/ehab892

[2] Roth GA, Mensah GA, Johnson CO, Addolorato G, Ammirati E, Baddour LM, Barengo NC, Beaton AZ, Benjamin EJ, Benziger CP, et al. Global burden of cardiovascular diseases and risk factors, 1990–2019: Update from the gbd 2019 study. J Am Coll Cardiol. 2020;76(25):2982–3021. 33309175 10.1016/j.jacc.2020.11.010 PMC7755038

[3] Winther S, Nissen L, Schmidt SE, Westra J, Andersen IT, Nyegaard M, Madsen LH, Knudsen LL, Urbonaviciene G, Larsen BS, et al. Advanced heart sound analysis as a new prognostic marker in stable coronary artery disease. Eur Heart J Digital Health. 2021;2(2):279–289. 10.1093/ehjdh/ztab031 PMC9707929 36712398

[4] Roy JK, Roy TS, Mukhopadhyay SC. Heart sound: Detection and analytical approach towards diseases. In: Modern sensing technologies. Cham: Springer; 2019. p. 103–145.

[5] Li S, Li F, Tang S, Xiong W. A review of computer-aided heart sound detection techniques. Biomed Res Int. 2020;2020: Article 5846191. 32420352 10.1155/2020/5846191 PMC7201685

[6] Kagiyama N, Shrestha S, Farjo PD, Sengupta PP. Artificial intelligence: Practical primer for clinical research in cardiovascular disease. J Am Heart Assoc. 2019;8(17): Article e012788. 31450991 10.1161/JAHA.119.012788 PMC6755846

[7] Zhao D, Liu J, Wang M, Zhang X, Zhou M. Epidemiology of cardiovascular disease in China: Current features and implications. Nat Rev Cardiol. 2019;16(4):203–212. 30467329 10.1038/s41569-018-0119-4

[8] Accountability Act. Health Insurance Portability and Accountability Act. Public Law. 2023.

[9] McMahan B, Moore E, Ramage D, Hampson S, Arcas BAY. Communication-efficient learning of deep networks from decentralized data. In: *Artificial intelligence and statistics*. Fort Lauderdale (FL): PMLR; 2017. p. 1273–1282.

[10] Xu J, Glicksberg BS, Su C, Walker P, Bian J, Wang F. Federated learning for healthcare informatics. J Healthc Inform Res. 2021;5(1):1–19. 33204939 10.1007/s41666-020-00082-4 PMC7659898

[11] Nguyen DC, Pham Q-V, Pathirana PN, Ding M, Seneviratne A, Lin Z, Dobre O, Hwang W-J. Federated learning for smart healthcare: A survey. ACM Comput Surv. 2022;55(3):1–37.

[12] Cheng K, Fan T, Jin Y, Liu Y, Chen T, Papadopoulos D, Yang Q. Secureboost: A lossless federated learning framework. IEEE Intell Syst. 2021;36(6):87–98.

[13] Liu Y, Fan T, Chen T, Xu Q, Yang Q. Fate: An industrial grade platform for collaborative learning with data protection. J Mach Learn Res. 2021;22(226):1–6.

[14] Chen T, Guestrin C. Xgboost: A scalable tree boosting system, *Proc ACM SIGKDD Int Conf Knowl Discov Data Min.* 2016:785–794.

[15] Das S, Pal S, Mitra M. Acoustic feature based unsupervised approach of heart sound event detection. Comput Biol Med. 2020;126: Article 103990. 32987200 10.1016/j.compbiomed.2020.103990

[16] Krishnan R, Rajpurkar P, Topol EJ. Self-supervised learning in medicine and healthcare. Nat Biomed Eng. 2022;6(12):1346–1352. 35953649 10.1038/s41551-022-00914-1

[17] Itahara S, Nishio T, Koda Y, Morikura M, Yamamoto K. Distillation-based semi-supervised federated learning for communication-efficient collaborative training with non-iid private data. IEEE Trans Mob Comput. 2021;22(1):191–205.

[18] Huang W, Li T, Wang D, Du S, Zhang J, Huang T. Fairness and accuracy in horizontal federated learning. Inf Sci. 2022;589:170–185.

[19] Qiu W, Quan C, Zhu L, Yu Y, Wang Z, Ma Y, Sun M, Chang Y, Qian K, Hu B, et al. Heart sound abnormality detection from multi-institutional collaboration: Introducing a federated learning framework. IEEE Trans Biomed Eng. 2024;1–12. 10.1109/TBME.2024.3393557 38700959

[20] Linardatos P, Papastefanopoulos V, Kotsiantis S. Explainable ai: A review of machine learning interpretability methods. Entropy. 2020;23(1):18. 33375658 10.3390/e23010018 PMC7824368

[21] Clifford GD, Liu C, Moody B, Springer D, Silva I, Li Q, Mark RG. Classification of normal/abnormal heart sound recordings: The PhysioNet/Computing in Cardiology Challenge 2016. In: Proc Comput Cardiol. p. 2016:609–2016:612.

[22] Liu C, Springer D, Li Q, Moody B, Juan RA, Chorro FJ, Castells F, Roig JM, Silva I, Johnson AE, et al. An open access database for the evaluation of heart sound algorithms. Physiol Meas. 2016;37(12):2181. 27869105 10.1088/0967-3334/37/12/2181 PMC7199391

[23] Lundberg SM, Lee S-I. A unified approach to interpreting model predictions. Adv Neural Inf Proces Syst. 2017;30:4765–4774.

[24] Zerka F, Barakat S, Walsh S, Bogowicz M, Leijenaar RT, Jochems A, Miraglio B, Townend D, Lambin P. Systematic review of privacy-preserving distributed machine learning from federated databases in health care. JCO Clin Cancer Inform. 2020;4:184–200. 32134684 10.1200/CCI.19.00047 PMC7113079

[25] Kaissis GA, Makowski MR, Rückert D, Braren RF. Secure, privacy-preserving and federated machine learning in medical imaging. Nat Mach Intell. 2020;2(6):305–311.

[26] Qiu W, Qian K, Wang Z, Chang Y, Bao Z, Hu B, Schuller BW, Yamamoto Y. A federated learning paradigm for heart sound classification. Paper presented at: 2022 44th Annual International Conference of the IEEE Engineering in Medicine & Biology Society (EMBC); 2022; Glasgow, Scotland, UK. 10.1109/EMBC48229.2022.9871319 36086612

[27] Liu JC, Goetz J, Sen S, Tewari A. Learning from others without sacrificing privacy: Simulation comparing centralized and federated machine learning on mobile health data. JMIR Mhealth Uhealth. 2021;9(3): Article e23728. 33783362 10.2196/23728 PMC8044739

[28] Qayyum A, Ahmad K, Ahsan MA, Al-Fuqaha A, Qadir J. Collaborative federated learning for healthcare: Multi-modal covid-19 diagnosis at the edge. IEEE Open J Comput Soc. 2022;3:172–184.

[29] Kumar Y, Singla R. Federated learning systems for healthcare: Perspective and recent progress. In: *Federated learning systems: Towards next-generation AI*. Cham: Springer; 2021. p. 141–156.

[30] Li J, Meng Y, Ma L, Du S, Zhu H, Pei Q, Shen X. A federated learning based privacy-preserving smart healthcare system. IEEE Trans Industr Inform. 2021;18(3):1–12.

[31] Tam P, Song I, Kang S, Kim S. Privacy-aware intelligent healthcare services with federated learning architecture and reinforcement learning agent. In: *International Conference on Computer Science and its Applications and the International Conference on Ubiquitous Information Technologies and Applications*. Singapore: Springer; 2022. p. 583–590.

[32] Chen Y, Qin X, Wang J, Yu C, Gao W. Fedhealth: A federated transfer learning framework for wearable healthcare. IEEE Intell Syst. 2020;35(4):83–93.

[33] Goetz J, Malik K, Bui D, Moon S, Liu H, Kumar A. Active federated learning. arXiv. 2019. 10.48550/arXiv.1909.12641

[34] Lin X, Chen H, Xu Y, Xu C, Gui X, Deng Y, Wang Y. Federated learning with positive and unlabeled data. In: *International Conference on Machine Learning*. Baltimore (MD): PMLR; 2022. p. 13344–13355.

[35] Jeong W, Yoon J, Yang E, Hwang SJ. Federated semi-supervised learning with inter-client consistency & disjoint learning. arXiv. 2020. 10.48550/arXiv.2006.12097

[36] Eyben F, Wöllmer M, Schuller B. Opensmile: The munich versatile and fast open-source audio feature extractor. Paper presented at: Proceedings of the 18th ACM international conference on Multimedia; 2010; Firenze, Italy.

[37] Eyben F, Weninger F, Gross F, Schuller B. Recent developments in opensmile, the Munich open-source multimedia feature extractor. Paper presented at: Proceedings of the 21st ACM International Conference on Multimedia; 2013; Barcelona, Spain.

[38] Eyben F. *Real-time speech and music classification by large audio feature space extraction*. Cham: Springer; 2015.

[39] Elkan C, Noto K. Learning classifiers from only positive and unlabeled data. Paper presented at: Proceedings of the 14th ACM SIGKDD International Conference on Knowledge Discovery and Data Mining; 2008; Las Vegas, NV, USA.

[40] Tao Y, Yang M, Li H, Wu Y, Hu B. Depmstat: Multimodal spatio-temporal attentional 531 transformer for depression detection. *IEEE Trans Knowl Data Eng*. 2024;36(7):2956–2966.

[41] Yang M, Wu Y, Tao Y, Hu X, Hu B. Trial selection tensor canonical correlation analysis (tstcca) for depression recognition with facial expression and pupil diameter. *IEEE J Biomed Health Inform*. 2023;1–12. 10.1109/JBHI.2023.3322271 37796673

